# Patient centered radiology: investigating 3 Tesla whole body MRI acceptance in cancer patients

**DOI:** 10.1007/s11547-023-01665-y

**Published:** 2023-07-03

**Authors:** Alice Rossi, Andrea Prochowski Iamurri, Danila Diano, Devil Oboldi, Emanuele Sintuzzi, Laghi Maurizio, Alice Andalò, Martina Cavallucci, Fabio Ferroni, Elena Amadori, Domenico Barone, Giuseppe Petralia

**Affiliations:** 1 Radiology Unit, IRCCS Istituto Romagnolo per lo Studio dei Tumori (IRST) “Dino Amadori”, Meldola, Italy; 2 Data Unit, IRCCS Istituto Romagnolo per lo Studio dei Tumori (IRST) “Dino Amadori”, Meldola, Italy; 3 Department of Oncology and Hemato-Oncology, University of Milan, Milan, Italy; 4 Precision Imaging and Research Unit, Department of Medical Imaging and Radiation Sciences, IEO European Institute of Oncology IRCCS, Milan, Italy

**Keywords:** WB-MRI, Oncology, Multiple myeloma, MRI, CT, PET/CT

## Abstract

**Introduction:**

Whole body magnetic resonance imaging (WB-MRI) is a promising emerging imaging technology for detecting bone and soft tissue pathology, especially in the onco-hematological field. This study aims to evaluate cancer patients' experience of WB-MRI performed on a 3T scanner compared to other diagnostic total body examinations.

**Material and method:**

In this prospective committee-approved study, patients completed a questionnaire in person (*n* = 134) after undergoing a WB-MRI scan to collect data on their physical and psychological reactions during the scan, the global satisfaction level, and preference for other types of MRI or computed tomography (CT), or positron emission tomography (PET/CT). Of all patients who had performed a CT or PET/CT the previous year, 61.9% had already undergone an MRI. The most common symptoms reported were: 38.1% perceived a localized increase in temperature and 34.4% numbness and tingling of the limbs. The scan time averaged 45 min and was well tolerated by most patients (112, 85.5%). Overall, WB-MRI was appreciated by the majority (121/134—90.3%) of patients who said they would probably undergo the procedure again. Patients preferred the WB-MRI in 68.7% of cases (92/134), followed by CT in 15.7% of cases (21/134) and by PET/CT in 7.4% (10/134), with 8.4% (11/134) of patients without any preference. The preference for imaging modalities was age-dependent (*p* = 0.011), while (*p* > 0.05) was independent of sex and a primary cancer site.

**Conclusion:**

These results demonstrate a high degree of WB-MRI acceptance from a patient's point of view.

**Supplementary Information:**

The online version contains supplementary material available at 10.1007/s11547-023-01665-y.

## Introduction

Whole body magnetic resonance imaging (WB-MRI) is a radiation-free imaging method, generally without contrast media administration, that detects and evaluates soft tissue and bone marrow pathology in different types of cancer. WB-MRI combines high-quality morphological sequences with “functional” images based on diffusion-weighted imaging (DWI), acquired at least from skull base to mid-thigh, following the well-established practices for whole body computed tomography (CT) and positron emission tomography/computed tomography (PET/CT). Other segments can be added to the basic protocol based on clinical need.

The core sequence of WB-MRI is DWI, which provides quantitative information on the diffusivity of water molecules by calculating the Apparent Diffusion Coefficient (ADC) [1, 2]. Numerous studies have demonstrated the capabilities of MRI for monitoring the response of bone metastases to treatment and the added value of WB-MRI sequences to overcome some of the limitations of standard imaging methods like CT, Bone Scintigraphy (BS), and 18F-Fluorodeoxyglucose (FDG) PET/CT in disease assessment and evaluation response to anticancer therapy [1–3]

Standard imaging methods have shown substantial limitations in the detection and assessment of bone disease, even though bone metastases from prostate and breast cancer are very common. [4, 5]. In response evaluation criteria in solid tumors (RECIST) version 1.1, bone metastases are considered non-measurable lesions [6]. Increased sclerosis and the sclerotic response of bone metastases can be difficult to distinguish with CT [5]. Bone Scintigraphy with technetium 99 m (99mTc)–methylene diphosphonate often results in underestimation of the disease extent, and difficulties in interpretation of serial bone scans are well described as “healing or flare response” of the bone [7].

High-quality morphological sequences are also useful in the detection, characterization, and response assessment of bone marrow. In this setting, T1-weighted images obtained with gradient-echo (GRE) Dixon acquisitions are usually performed to generate in- and opposed-phase images, permitting the calculation of fat-only and water-only images as well as the relative Fat Fraction map (rFF%) [8].

Some parameters of WB-MRI, in particular values from the Apparent Diffusion Coefficient (ADC) map and the relative Fat Fraction (rFF%) map, are emerging as possible MR imaging biomarkers at diagnosis and during therapy response [9, 10].

The development of WB-MRI has led to a new dimension for evaluating bone marrow involvement in patients affected by immune plasma cell disorders. For the assessment of bone disease, the International Myeloma Working Group (IMWG) suggests a variety of imaging assessments, including low-dose CT, (FDG)PET/CT, and MRI. Given WB-MRI's high sensitivity, it has been recommended for patients affected by bone solitary plasmocitoma and for patients suspected of having non-IgM monoclonal gammopathy of undetermined significance (MGUS), smouldering multiple myeloma or multiple myeloma and who underwent low-dose WB-CT or (FDG)PET/CT with negative or inconclusive findings [11, 12].

Moreover, according to the European Union directive 2013/59/Euratom, concerns have arisen over radiation exposure from imaging examinations, which has led to the E.U. expressing interest in WB-MRI as an alternative to standard imaging procedures, especially in young cancer patients with a long-life expectancy.

As a result, WB-MRI is currently implemented in several professional society guidelines, including advanced prostate cancer [13, 14], multiple myeloma [11], and cancer screening in a high-risk population for genetic predisposition [15]. A recent expert review panel [16] found a consensus supporting WB-MRI as a first-line imaging approach in patients with multiple myeloma and pregnancy, as well as a well-established screening tool in patients with cancer risk factors (strong evidence for the Li-Fraumeni syndrome) [17], and also useful in assessing patients with known bone and visceral metastases [16]. Moreover, WB-MRI has a key role in patients affected by known metastatic disease, non-avid FDG lymphomas, or bone neoplasm. Finally, there is an increasing number of studies that report its use for cancer screening in the general population [18]

However, MRI is generally considered more stressful than other imaging modalities [19, 20], such as CT and PET/CT; due to its longer acquisition times, WB-MRI could be even more stressful, thus jeopardizing the image quality and discouraging the adoption in clinical routine. In particular, compared to the other whole body imaging modalities, acquisition times of WB-MRI are longer: a complete WB-MRI takes around 35–50 min, in which the patient should lie still, tightly wrapped in the body coils, inside a narrow tube with a loud background noise.

In general, there is no evidence of negative effects associated with performing MRI on 1.5T or 3T scanners, but magnetic field strength was found to be statistically associated with new-onset dizziness; Weintraub et al. found that most patients of subjects (86%) did not notice a difference between 1.5T and 3T scan but a 14% of subjects experienced sensory stimulation (new or altered symptoms) in both 3T and 1.5T units, predominantly in women, indicating the existence of a threshold of magnetic susceptibility and sexual vulnerability [21].

WB-MRI is usually used on cancer patients, who are often older than the general population, have age-related disabilities, are more likely to develop painful bone metastases, and are subjected to higher physiological pressures. All of these factors can affect a patient's ability to perform a WB-MRI scan.

The effects of the interaction between patients and imaging are often overlooked in favor of evaluating diagnostic efficacy, and, despite the improvements, there is the possibility that imaging creates a major psychological and physical burden especially on cancer patients. As various whole body imaging modalities can be used for diagnosis and follow-up in different settings, understanding the patient experience may be beneficial in particular in modern healthcare where it is crucial to recognize and meet patients' needs and preferences [22]. To guarantee patient-centered care and good-quality scans, acceptance of WB-MRI is a pivotal topic and the studies available yielded somewhat divergent findings but none of them was performed exclusively on 3T MRI scanners.

Our study aimed to evaluate the patient experience and acceptability of a 3T WB-MRI in an oncological setting also with a comparison with PET/TC or Contrast Enhancement CT (CE-CT).

## Materials and methods

This prospective study was approved by the Ethical Committee (IRST100.15), and all participants signed an informed consent to be enrolled in the study, in addition to the standard consent required by law for MRI scans.

From October 2020 to March 2022, all the patients who underwent a first-in-life WB-MRI at our institution for clinical reasons (cancer staging or follow-up) were asked to participate in this study for their feedback on the procedure, even if they did not complete the scan.

The patients completed the questionnaire under the Radiologist’s supervision after the WB-MRI before leaving the MRI site. The questionnaire was divided into several parts: the first included demographic information and the examination date.

The second part concerned the patient’s experience: the physical and psychological reactions during the scan, such as dizziness, involuntary muscle contraction, tingling, tickles, increased temperature, sweating, fatigue, fear, headache, nausea, and tachycardia. All the data were evaluated using a four-point scale (0 not present; 1 present; 2 unpleasant, but tolerable; 3 intolerable).

The third part evaluated the global level of satisfaction of WB-MRI considering exam duration, noise, the narrowness of the tube, and comfort of positioning (1 at ease; 2 low uneasiness; 3 moderate uneasiness; 4 strong uneasiness). Finally, in patients who had already performed other total body examinations, preference between WB-MRI and other diagnostic modalities was investigated.

### WB-MRI protocol at our institution

Patient candidates for WB-MRI are carefully evaluated and selected by the prescribing physician. He gives potential candidates an in-depth explanation of the technique and possible benefits on patient management deriving from the diagnostic result of this method; informed consent is acquired. Moreover, patient characteristics may influence the exam modality. For example, patients with claustrophobia or MRI-unsafe devices must undergo a CT or PET/CT and are excluded. In case of pain or anxiety, appropriate therapy is given. Immediately before the exam, the radiologist explains the exam procedure to the patients again and administers additional medication if needed to alleviate pain and anxiety.

All the WB-MRI were performed on a 3T MRI scanner (Ingenia Philips, Eindhoven, Netherlands).

A head/neck helmet-like coil and a flexible surface body coil are applied to patients for WB-MRI scans.

Both earplugs and earmuffs are put on patients, and the patient's favorite music is played.

Fasting is not required for WB-MRI without intravenous contrast media, but patients are advised to have only a light meal before the exam.

The WB-MRI basic protocols at our institution are based on MET-RADS [23] and MY-RADS [24] and consist of sagittal T1-weighted and sagittal STIR T2-weighted sequences of the entire spine, axial diffusion-weighted whole body imaging with background body signal suppression (DWIBS) technique at two b-Value (b50 e b800), axial GRE T2 and axial T1 Gradient Echo (GRE) mDixon sequences from vertex to mid-thighs (from vertex to knees for multiple myeloma). From the DWI and the Dixon-type images, the Apparent Diffusion Coefficient (ADC) map and the relative Fat Fraction (rFF%) maps are, respectively, reconstructed,

Usually, patients are prepared for the intravenous contrast medium only in case of known or suspected brain metastasis, according to clinical needs, particularly in patients with melanoma [25]. Dedicated brain assessment is performed with axial FLAIR, pre- and post-contrast 3D T1W GRE.

**Fig. 1:**
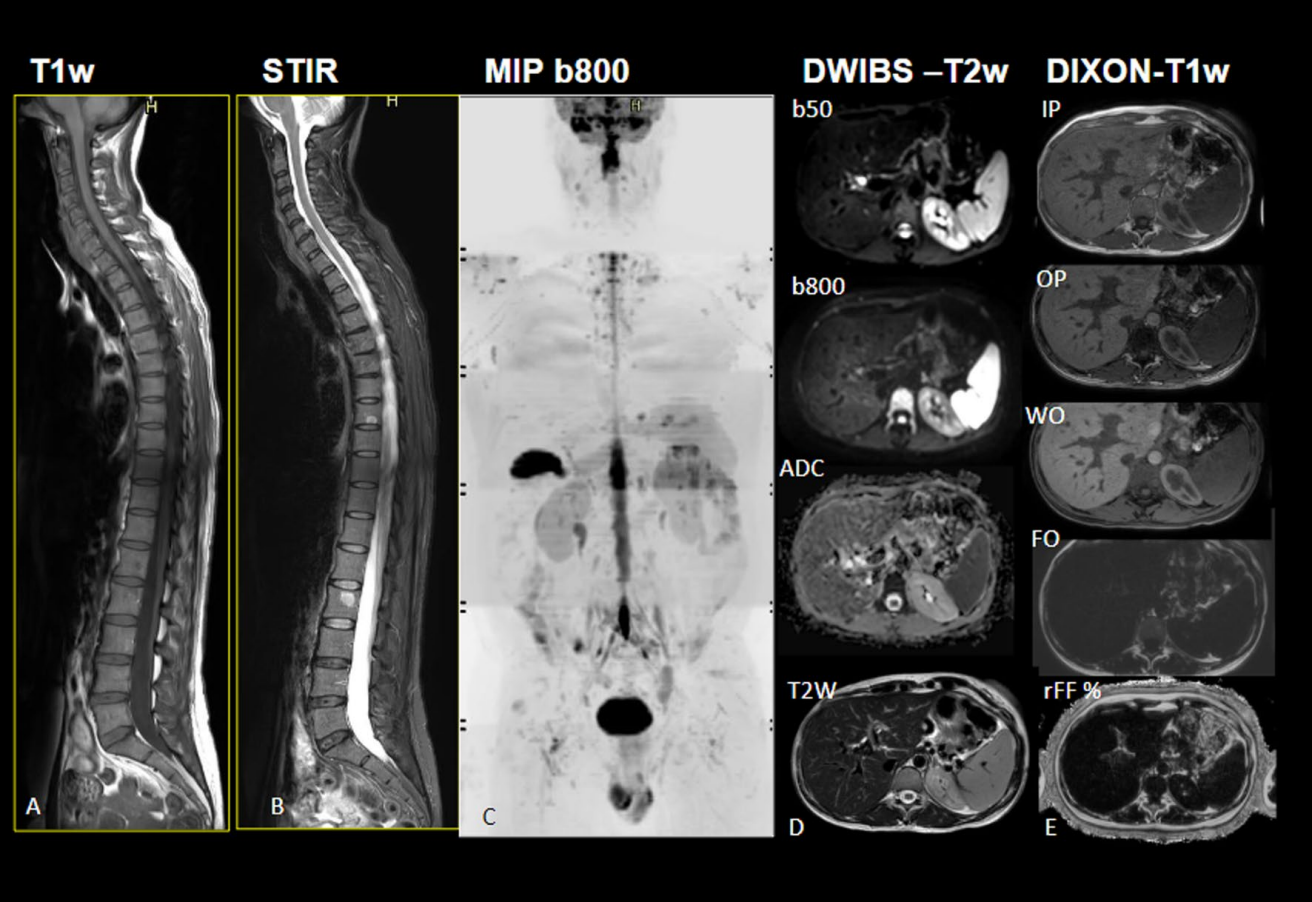
Images were acquired according to our WB-MRI protocols

At the time of writing, the ONCO-RADS [26] acquisition protocol was used only for some very young patients affected by metastatic paraganglioma/pheochromocytoma in follow-up after therapy due to radiation protection. Detailed parameters of the most common protocols used at our institution are described in Table 1 whereas the sequence parameters are described in Table 2. An example of images acquired is shown in Fig. 1.

**Table 1:** Acquisition protocols of WB-MRI at our institution

Sequence description	Multiple myeloma [16] (MY-RADS)	Metastatic prostate cancer [15] (MET-RADS-P)	Suggested protocol breast cancer	Suggested protocol melanoma
Whole spine sagittal T1 W TSE 4 mm	Yes	Yes	Yes	Yes
Whole spine sagittal T2 W TSE STIR 4 mm	Yes	Yes	Yes	Yes
Whole body axial T1W GRE mDIXON-XD 3 mm	Yes (from vertex to knees)	Yes (from vertex to mid-thighs)	Yes (from vertex to mid-thighs)	Yes (from skull base to mid-thighs)
Whole body axial DWI 6 mm b50 e b800 s/mm2@-ADC map@-3D-MIP reconstructions of b800 images	Yes (from vertex to knees)	Yes (from skull base to mid- thighs)	Yes (from skull base to mid- thighs)	Yes (from skull base to mid- thighs)
Whole body axial T2W TSE 5 mm	Yes (from skull base to mid- thighs)	Yes (from skull base to mid- thighs)	Yes (from skull base to mid- thighs)	Yes skull base to mid- thighs
Brain assessment			optional Brain: FLAIR axial, pre e post-contrast 3D T1W GRE at 1 mm	Yes Brain: FLAIR axial, pre e post-contrast 3D T1W GRE at 1 mm

**Table 2:** Acquisition parameters of the WB-MRI sequences at our institution

Philips Ingenia 3T (bore diameter 70 cm)	DWIBS	T1 (spine)	T2 STIR (spine)	T1 mDIXON-XD FFE (3D)	T2 multivane + trigger
Imaging plane	Axial	Sagittal	Sagittal	Axial	Axial
Field of view (cm)	450 × 400	200 × 350	200 × 350	430 × 430	430 × 430
Matrix size	128 × 112	224 × 346	224 × 346	268 × 217	288 × 288
Repetition time (ms)	4737 (shortest)	680–700	3400–5000	3.8 (shortest)	2014 (shortest)
Echo time (ms)	62 (shortest)	10	60	1.31 (shortest)	126 (shortest)
Fast imaging/factor	EPI factor 47	TSE factor 4	TSE factor 17	none	TSE factor 50/MV perc. 240%
Parallel imaging factor	2.4	2	2	3	2.9
No. of signals averaged for high b-value images/NSA	1 (b = 50), 6 (b = 800)	1	1	1	1
Section thickness (mm)	6	4	4	3	5
Gap (mm)	1	0.4	0.4	0	0.5
Voxel (mm)	3.5 × 3.5 × 6	0.9 × 1 × 4	0.9 × 1 × 4	1.6 × 1.97 × 3	1.5 × 1.5 × 5
Slices	30	18	18	83	48
Direction of motion probing gradients/gradient mode	Enhanced	Default	Default	Maximum	Maximum
Receiver bandwidth	3181.2 Hz	290.6 Hz	267.6 Hz	1504.4 Hz	438.4 Hz
Fat suppression	STIR (TI = 220 ms)	None	STIR (TI = 200)	Dixon	None
b-values (s/mm^2^)	50 and 800	0	0	0	0
Acquisition time per station	5 min 13 s	3 min 20 s	3 min 55 s	14 s (breath hold)	≃ 3 min

The imaging protocol can also be “tailored” depending on clinical needs, adding other specific imaging targets.

### Statistical analysis

Statistical analyses were performed using the statistical and data management package MedCalc for Windows (Version 5.0.1.0 Ostend, Belgium). ANOVA or Pearson's Chi-squared test was performed to investigate the influence of age (ANOVA), sex (Chi-squared), and primary cancer (Chi-squared) on the acceptance of WB-MRI compared to other total body imaging modalities (CT or PET). Statistical significance was set at *P* < 0.05 for all tests. Descriptive data are reported as frequencies or mean and standard deviation (± s.d.).

**Fig. 2:**
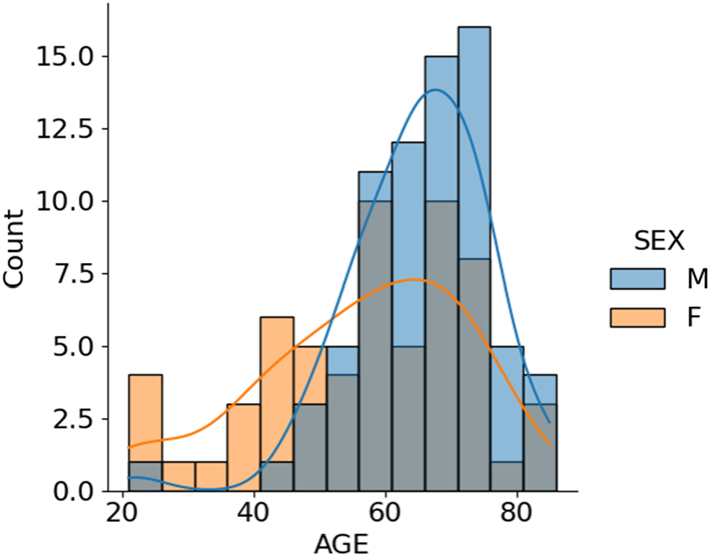
Age distribution divided by patient's gender

A total of 134 patients (73 males, 61 females) with a mean age of 61.3 ± 13.8 years were enrolled in this study (Fig. 2).

Multiple myeloma (56/134—41.8%) and prostate cancer (20/134—14.9%) were the most common cancer types among patients, followed by melanoma (16/134—11.9%) and breast cancer (14/134—10.4%); all the other patients included in the study are shown in Table 3.

**Table 3:** Primary cancer type of patients included in the study

Primary cancer	n—%
*Multiple myeloma*	56/134—41.8%
*Prostate cancer*	20/134—14.9%
*Melanoma*	16/134—11.9%
*Breast cancer*	14/134—10.4%
*Neuroendocrine tumors*	7/134—5.2%
*Lymphoma*	6/134—4.5%
*Ovarian cancer*	5/134—3.7%
*Renal cancer*	3/134—2.2%
*Gastric cancer*	2/134—1.5%
*Thyroid Cancer*	2/134—1.5%
*Thymoma*	1/134—0.7%
*Gastrointestinal stromal tumor*	1/134—0.7%
*Pheochromocytoma*	1/134—0.7%

All patients had already performed other total body imaging techniques the previous year (CT and PET/CT); 83 patients (61.9%) had already performed MRI in different body districts with a similar scanner 1.5T or 3T, and only one patient had already undergone WB-MRI in another hospital on 1.5T scanner.

In response to an open-ended question, none of these patients reported differences in symptoms or approval compared to previous MRIs.

**Fig. 3:**
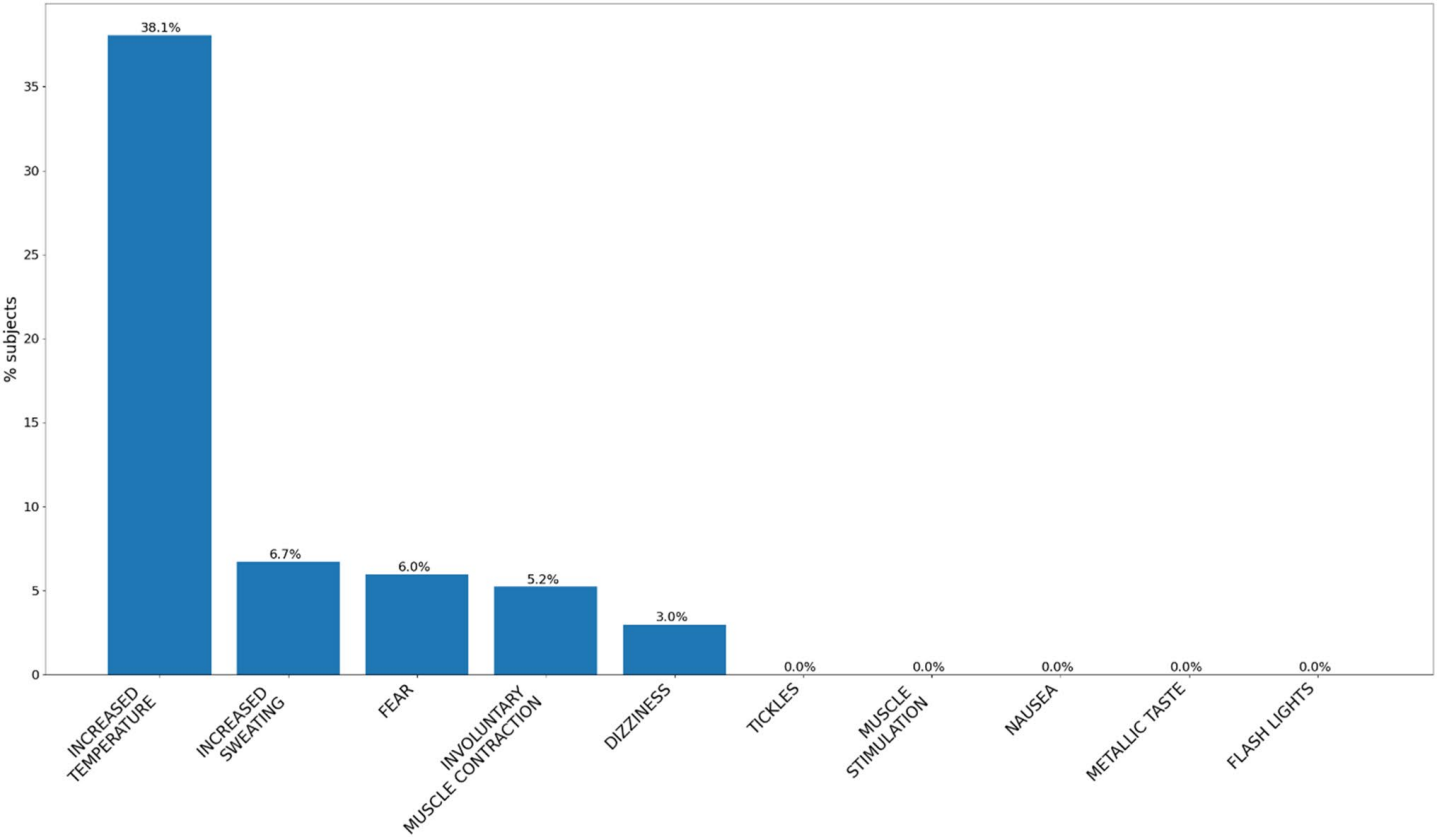
Percentage of symptoms recorded during the examination

The most frequent symptom reported by the patients was a localized increase in temperature (51/134—38.1%), which was deemed tolerable in all cases, whereas sweating was reported in only nine cases (9/134—6.7%) and always tolerable (Fig. 3). Numbness and tingling of the limbs were both reported in 46 (46/134—34.4%) cases and were deemed unpleasant or intolerable in 9 (9/46—19.6%) and 3 (3/46—6.5%) cases, respectively.

Patients reported involuntary muscle movements and dizziness in 7/134 (5.2%) and 4/134 (3%) cases. Eight patients experienced fear (8/134—6.0%) at the beginning of the procedure but disappeared throughout the examination. Moreover, two patients (2/134—1.5%) described palpitations.

There were no reports of any additional symptoms, including nausea, metallic taste, headaches, tickles, scotomas, or muscle stimulation.

**Fig. 4:**
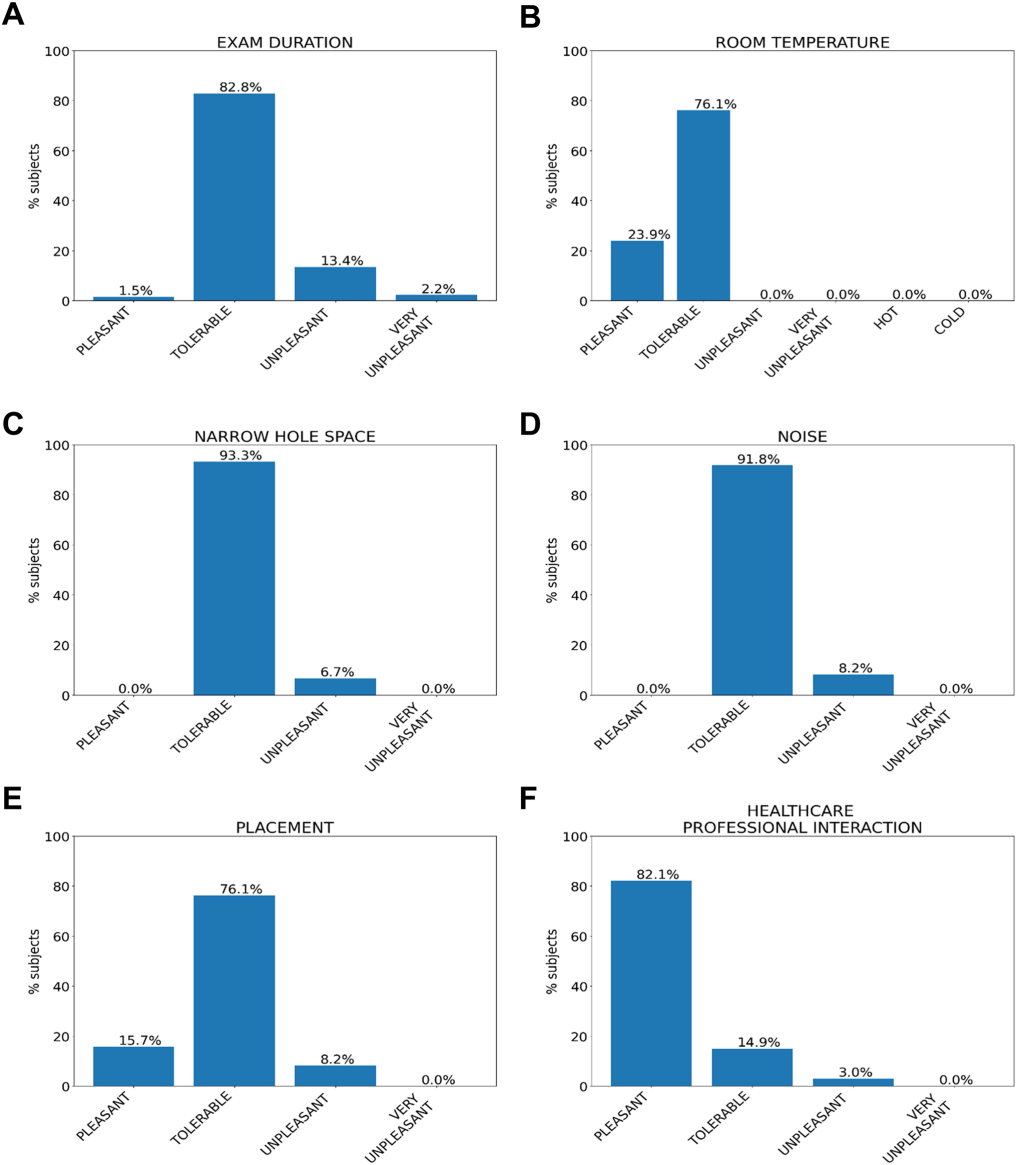
Figure shows the detection features of the Whole Body exam based on the level perceived by the patient. The six graphs show the percentage of patients divided according to the level of exam characteristics recorded. The Whole-Body exam features studied were: exam duration graph (**A**), room temperature for graph (**B**), narrow hole space graph (**C**), noise graph (**D**), placement graph (**E**), and healthcare professional interaction in graph (**F**)

Most patients were able to tolerate the length of the WB-MRI as well as the noise and positioning. Only a few patients found the length of the examination, confined space, and positioning to be uncomfortable. In particular, 113 patients (113/134 84.3%) perceived the WB-MRI duration as tolerable, 21 patients (21/134—15.7%) as unpleasant, and three patients considered it intolerable. Additionally, 26 patients (26/134—19.4%) considered the examination tiring and 15 out of 26 said it was very exhausting (Fig. 4). Bore narrowness, noise, and positioning were considered unpleasant, respectively, by 9/134 (6.7%), 11/134 (8.2%), and 11/134 (8.2%) of patients, but never intolerable.

Overall, WB-MRI was appreciated by the vast majority of patients (121/134—90.3%) who said they would probably undergo the procedure again, whereas 13/134 (9.7%) said they would not repeat the examination in the future due to the narrowness of tube (3/13), lengthy examination time (8/13), and uncomfortable positioning (2/13). When asked to express a preference between the whole body imaging modalities, patients preferred the WB-MRI in 68.7% of cases (92/134), followed by Computed Tomography in 15.7% of cases (21/134) and Positron Emission Tomography in 7.4% (10/134), with a 8.2% (11/134) of patients who said they did not have any preference.

The preference for imaging modalities was influenced by age (*p* = 0.011) with younger patients preferring WB-MRI, independently of sex and primary cancer (*p* > 0.05).

## Discussion

Patient acceptability of new technologies is crucial for developing and implementing imaging methods and obtaining successful diagnostic examinations.

Low patient acceptability may lead to a lack of compliance during image acquisition, which can negatively affect the image quality [27] due to motion artifacts, especially if the examination requires a long acquisition time. Our study showed that most cancer patients of all types accept and highly appreciate WB-MRI compared to the other total body imaging modalities.

The most common symptom reported by patients in our study was a localized increase in temperature, most likely due to the high overall Specific Energy Dose (SED) of WB-MRI, which implies an increase in body temperature, sometimes associated with sweating. Thus, maintaining a medium–high ventilation level inside the bore, allowing optimal heat dissipation, is crucial.

Numbness and tingling are two more common symptoms reported by patients, which may be connected to the patient's positioning and the requirement to keep motionless. The proper acquisition of MRI images is heavily reliant on the patient's cooperation, which is more evident in WB-MRI, given the lengthy examination time, and the concatenation of sequences, which are very sensitive to voluntary and involuntary movements: It is thus critical to position the patient appropriately and comfortably, for example, with a knee cushion and armrest if available, to prevent patient movement.

The length of the examination and the need to concentrate to keep motionless following the operator's orders contribute to making the examination tiring, which is another frequent complaint made by patients; however, in our study, most of the patients that considered tiring (16/26—61.5%) or lengthy (13/21—61,9%) the examination will undergo again to WB-MRI, and some of them (9/26 and 5/21) prefer the WB-MRI to the other whole body imaging modalities, mostly due to the absence of ionizing radiations.

The examination's noise level was another issue raised frequently by patients. The loudness of several sequences—most notably DWI and T1W GRE mDixon—is quite high, and wearing both earplugs and earmuffs improve patient acceptability of the examination by reducing the perceived acoustic noise and enabling operator-patient communication, such as during breath-hold commands. Additionally, several patients suggested that listening to music could help reduce the discomfort induced by the loudness of the scan.

MRI scanners are notoriously small, and the mere thought of facing an MRI can generate anxiety in some patients. Consequently, identifying patients with claustrophobia and speaking to them beforehand are fundamental. To alleviate MRI-related anxiety, the patient is asked to keep their eyes closed for the duration of the procedure; in mildly claustrophobic individuals, a dark blindfold can be helpful.

The patients' comments highlight the importance of the staff's involvement in explaining the procedure by assisting the patient in coping with intrinsic problems of WB-MRI to complete the examination and obtain reliable images. Furthermore, careful patient selection based on clinical circumstances is essential, making collaboration with oncologists even more critical.

This study also found that many patients prefer WB-MRI over other whole body imaging modalities, such as PET/CT and CT, since it is contrast-free and ionizing radiation-free, despite lasting longer, loudness, complete body and head immersion inside a tight "tube," and usage of receiver coils.

To our knowledge, only a few studies have investigated the patient experience of WB-MRI and compared it to other total body imaging modalities; they have shown some results on the acceptance of WB-MRI performed on 1.5T scanners, but none have focused on 3T scanners alone.

In our experience, the majority (90.3%) of patients appreciated WB-MRI and said they would probably undergo the procedure again. These data are in line with the studies by Adams et al. [28] and Oliveri et al. [29] in cancer patients and with those of Busacchio et al. [30] in self-referring asymptomatic subjects screening. In these studies, the WB-MRI performed on 1.5T scanners was well-accepted and considered more tolerable than other total body imaging modalities by most patients.

They discovered that a higher WB-MRI load was related to significant distress and comorbidities. In the experience of patients who had WB-MRI for screening, 98.5% felt high to a very high degree of usefulness, and 95.4% would repeat the examination [30].

Oliveri et al. [29] found that the level of WB-MRI acceptance was > 81% high or higher. Adam et al. found that WB-MRI was less concerning than CT, less unpleasant, and finally, patients felt better after WB-MRI.

Evans et al. [31, 32] investigated patient experience and acceptability of WB-MRI compared to standard staging imaging methods in patients affected by lung or colon rectal cancer enrolled in the Streamline line L and the Streamline line C trials, respectively. The overall satisfaction was lower for WB-MRI than PET/CT and, above all, compared to CE-CT. They also noted that WB-MRI is generally perceived as more stressful than other imaging techniques partly because of the potential, perceived by patients, to be able to detect a cancer diagnosis or additional elements associated with already known diagnoses that may result in a worse prognosis and different treatment implications (viewed both positively and negatively).

According to the study of Dyrberg et al. on the pleasure of WB-MRI in prostate cancer patients, WB-MRI had a satisfactory overall enjoyment level [33].

The Ryder A et al. study regarding patients affected by Myeloma demonstrated a high level of the overall satisfaction for both WB-MRI and other methods [34].

Our study and previous research showed that duration and noise are factors that negatively affect the WB-MRI experience, particularly in patients with pulmonary symptoms, pain, and/or claustrophobia, whereas staff support and information, as well as comfort during acquisition (posture and mental relaxation also through listening to music), are factors that increase the overall enjoyment of the method. Additionally, psychological assistance and relaxation techniques may be helpful.

Scan acceptability is greater in younger patients probably due to awareness of the long-life expectancy and risks associated with the use of ionizing radiation also known in the general population.

One potential limitation of this study is that even if we asked all patients who had undergone the WB-MRI to participate in the study, all those who interrupted the procedure refused to fill the questionnaire. Thus, the experience and acceptance of the techniques in this study population were probably more favorable compared to a background population. Another limitation of the study is that it did not investigate the relationship between acceptability and pantient’s level of education.

## Conclusions

Our study reveals that WB-MRI exams are well-accepted by adult patients suffering from various types of malignancies and bone disorders. This is a good starting point when thinking about how to apply the technology in clinical practice because WB-MRI seems to be just as pleasant as other total body imaging modalities (PET/CT, CE-CT).

Evaluating a new imaging method through the eyes of the patients is an important factor in successfully developing and implementing WB-MRI in clinical practice. This analysis is essential to make our procedures more patient-friendly and offer a more patient-centered approach.

## Supplementary Information

Below is the link to the electronic supplementary material.

Supplementary file1 (XLSX 24 KB)
